# The chicken or the egg? A case of sympathetic staphylococcal empyema thoracis and splenic abscess

**DOI:** 10.1093/jscr/rjaa541

**Published:** 2021-11-11

**Authors:** Kudzayi H Kutywayo, Joyce Thekkudan, Mohammed Fiyaz Chowdhry

**Affiliations:** Department of Thoracic Surgery, Glenfield Hospital, Leicester, UK; Department of Thoracic Surgery, Glenfield Hospital, Leicester, UK; Department of Thoracic Surgery, Glenfield Hospital, Leicester, UK

## Abstract

Multicavitary abscesses are uncommon in immunocompetent individuals. Splenic abscesses being relatively uncommon with an incidence of 0.1–0.7% in quoted series [ 1]. Commonly reported cases are secondary infective endocarditis. To have both a splenic abscess and empyema concurrently is rare. We describe a case of a patient with a large left-sided empyema thoracis and concurrent splenic abscess.

## CASE REPORT

**Figure 1:**
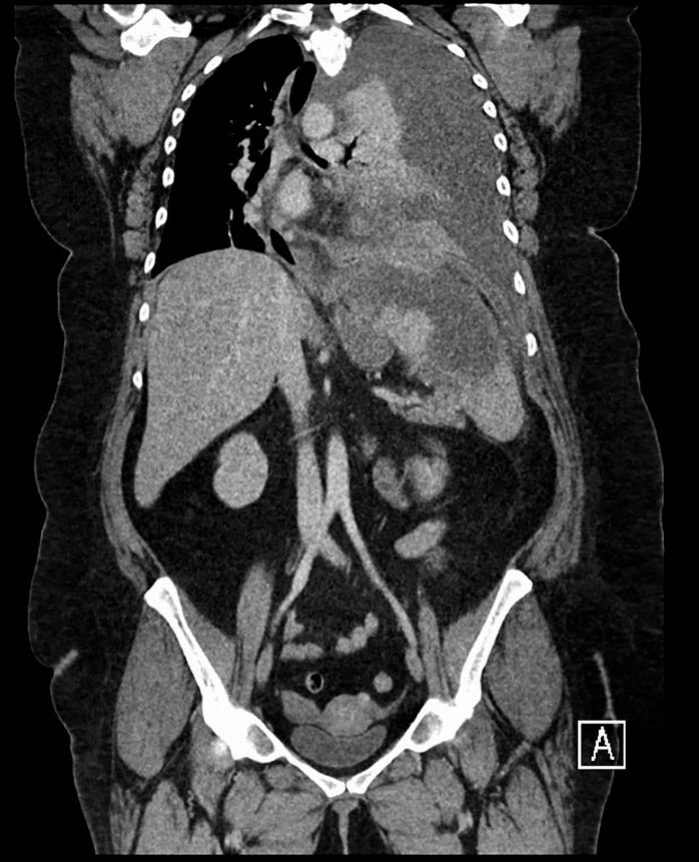
CT chest, abdomen and pelvis coronal view: left-sided empyema and splenic abscess.

**Figure 2:**
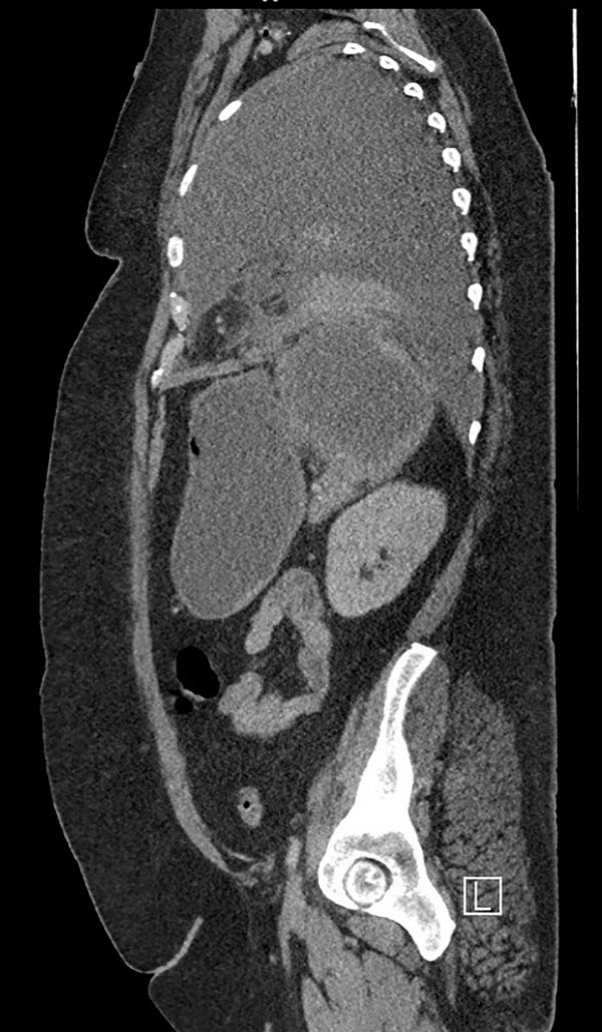
CT chest, abdomen and pelvis sagittal view: left-sided empyema and splenic abscess.

Forty-four-year-old clinically obese woman whose only past medical history was mild asthma with no use of systemic steroids. Initially presented with parapneumocic effusion following a recent bout of pneumonia which was not amenable to aspiration. Prior to this, the patient had experienced vague abdominal symptoms 1 month prior to the onset of the pneumonia. She was treated with oral amoxicillin. Twenty-five days later, she presented again in the Accident & Emergency Department with left-sided chest pain, left flank pain associated with fever and worsening dyspnea. Computed tomography (CT) scan (Fig. 1) showed a significant left-sided effusion with a complete collapse of the left lung and mild mediastinal shift towards the right. A 12.5 cm collection in the upper pole of the spleen was noted which appeared to extend through the diaphragm (Fig. 2). She was started on the sepsis pathway as per hospital protocol. A seldinger chest drain was inserted which immediately drained 1500 ml of pus. She underwent a thoracoscopic washout, debridement and decortication. The chest drains were removed 9 days post-operatively. She was transferred to General surgeons where a percutaneous drain (pigtail catheter) was inserted under ultrasound guidance. Three hundred and five milliliters in a total of pus was drained from the splenic abscess. *Staphylococcus aureus* was isolated from both the pleural and splenic collection. Blood cultures and transthoracic echocardiogram were unrevealing. A blood bourne virus screen (Hepatitis B, Hepatitis C, HIV) was negative as well. A repeat ultrasound scan showed complete resolution of the splenic abscess with normal splenic architecture.

## DISCUSSION

A timeline of events to establish whether the empyema led to the splenic abscess or vice versa is still unclear. The commonest source of splenic abscesses is a cardiac nidus [2]. No echocardiographic signs of infective endocarditis were noted in the patient. Could this be due to the timing of the echo (already 2 weeks of intravenous antibiotics had been given) or the lower sensitivity of a transthoracic echocardiogram when compared to a transoesophageal echo (TTE 69.4% vs TOE 93.3%) [3]? Increased BMI also presents a technical challenge as the increased layer of adipose tissue causes an attenuation of the ultrasound signal. Failure to identify a cardiac focus as the source of the splenic abscess, it would seem reasonable to posit a pulmonary source.

Anatomically the spleen lies in close relation with the left hemidiaphragm. Posteriorly, the endothoracic fascia and transversals fascia are continuous and can act as a route of tracking pus or fluid transdiaphragmatically [4].

Case reports of transdiaphragmatic fistulas connecting subphrenic collections and empyemas are uncommon [5]. Could the culprit responsible for dissemination of infection from the abdominal to pleural cavity (or vice versa) be a splenothoracic fistula [6]? A communication between the two collections was highlighted in the first CT scan. Staphylococcal species with similar sensitivities were isolated on cultures from both compartments.

Regardless of origin, bicompartmental concurrent infections of the thoracic and abdominal cavity though uncommon are still a recognized cause of morbidity.
